# Structural variation and missense mutation in *SBDS* associated with Shwachman-Diamond syndrome

**DOI:** 10.1186/1471-2350-15-64

**Published:** 2014-06-04

**Authors:** Claudia M B Carvalho, Luciana W Zuccherato, Christopher L Williams, Nicholas J Neill, David R Murdock, Matthew Bainbridge, Shalini N Jhangiani, Donna M Muzny, Richard A Gibbs, Wan Ip, Robert Paul Guillerman, James R Lupski, Alison A Bertuch

**Affiliations:** 1 Department of Molecular and Human Genetics, Baylor College of Medicine, Houston, TX, USA; 2 Centro de Pesquisas René Rachou – FIOCRUZ, Belo Horizonte, MG, Brazil; 3 Human Genome Sequencing Center, Baylor College of Medicine, Houston, TX, USA; 4 Department of Pediatrics, Baylor College of Medicine, Houston, TX, USA; 5 Texas Children’s Hospital, 1102 Bates, FC 1200, Houston, TX 77030, USA; 6 Program in Physiology and Experimental Medicine, The Research Institute, The Hospital for Sick Children, Toronto, Ontario, Canada; 7 Department of Radiology, Baylor College of Medicine, Houston, TX, USA

**Keywords:** Shwachman-Diamond syndrome, *SBDS*, Structural variation, Genomic rearrangement, Non-allelic homologous recombination, Low copy repeat, Whole exome sequencing, Copy number variation, Recessive disease

## Abstract

**Background:**

Shwachman–Diamond syndrome (SDS) is an autosomal recessive ribosomopathy caused mainly by compound heterozygous mutations in *SBDS*. Structural variation (SV) involving the *SBDS* locus has been rarely reported in association with the disease. We aimed to determine whether an SV contributed to the pathogenesis of a case lacking biallelic *SBDS* point mutations.

**Case presentation:**

Whole exome sequencing was performed in a patient with SDS lacking biallelic *SBDS* point mutations. Array comparative genomic hybridization and Southern blotting were used to seek SVs across the *SBDS* locus. Locus-specific polymerase chain reaction (PCR) encompassing flanking intronic sequence was also performed to investigate mutation within the locus. RNA expression and Western blotting were performed to analyze allele and protein expression. We found the child harbored a single missense mutation in *SBDS* (c.98A > C; p.K33T), inherited from the mother, and an SV in the *SBDS* locus, inherited from the father. The missense allele and SV segregated in accordance with Mendelian expectations for autosomal recessive SDS. Complementary DNA and western blotting analysis and locus specific PCR support the contention that the SV perturbed SBDS protein expression in the father and child.

**Conclusion:**

Our findings implicate genomic rearrangements in the pathogenesis of some cases of SDS and support patients lacking biallelic *SBDS* point mutations be tested for SV within the *SBDS* locus.

## Background

Shwachman Diamond syndrome (SDS; OMIM 260400) is a rare autosomal recessive condition characterized by bone marrow dysfunction, typically neutropenia that can progress to aplastic anemia; and pancreatic exocrine insufficiency, which may improve beyond early childhood [1]. Skeletal abnormalities, such as metaphyseal chondrodysplasia and thoracic dystrophy, and short stature are also common. *SBDS*, located at 7q11.21, is the only gene associated with SDS to date [2]. Compound heterozygous mutations in *SBDS* can be identified in 75% to 98% of SDS patients [3,4]. The majority of mutated *SBDS* alleles, 74% in the pioneering study [2], are the result of recurrent gene conversion events between *SBDS* and the *SBDSP1* pseudogene, which shares 97% nucleotide sequence identity with *SBDS* and resides in an inverted orientation at a locally duplicated genomic segment, which maps ~5.8 Mb in the telomeric direction. This inverted repeat genomic architecture is predicted to result in genomic instability by providing substrates for non-allelic homologous recombination (NAHR) and inversion structural variation (SV) [5-7]. The NAHR hypothesis is further supported by the high frequency that disease-associated alleles result from apparent gene conversion events, likely reflecting alternate resolution of a Holliday structure. Nevertheless, large structural variants have been rarely implicated in the pathogenesis of the disease; in fact, only a paternally inherited *Alu*-mediated deletion that removes exon 3 and part of flanking introns has been reported thus far [8].

*SBDS* is comprised of 5 exons, spanning 7.9 kb, has a 1.6 kb transcript and encodes a 250 amino acid protein, involved in ribosome biogenesis [2,9,10]. The two recurrent gene conversion mutations encode either a frameshift (p.84Cfs3) or nonsense (p.K62X) mutation [2]. Patients carrying compound heterozygous nonsense and/or frameshift mutations lack detectable SBDS protein by immunoblotting using SBDS antibody raised against the carboxy-terminal peptide [10]. Importantly, patients carrying compound heterozygous mutations, in which one leads to protein truncation and the other is a missense mutation, demonstrate markedly decreased SBDS protein expression in comparison to unaffected family members that carry only one heterozygous mutation. The SBDS protein expression in heterozygous carriers is often comparable to family members carrying non-altered *SBDS* alleles [11]. Thus, it has been suggested that the SDS disease phenotype is a consequence of expression of hypomorphic *SBDS* alleles [12].

We investigated a patient with SDS with severe disease manifestations who carried a single novel *SBDS* missense mutation. We uncovered an SV on the remaining allele, which resulted in altered *SBDS* expression and markedly decreased SBDS protein.

## Case presentation

### Methods

#### *Research subjects*

The proband with SDS was ascertained at the Texas Children’s Hospital Hematology Center. Informed consent for participation, sample collection and analysis was obtained using protocols H-17698 and H-23793, approved by the Institutional Review Board for Baylor College of Medicine and affiliated hospitals.

#### *Lymphoblastoid cell lines*

Epstein-Barr virus-transformed lymphoblastoid cell lines (LCLs) were generated from the proband (BAB3762), her parents (BAB3763 - mother, BAB3764 - father) and siblings (unaffected full sisters BAB5568, BAB5569, and BAB5570) by the Tissue Culture Core within the Intellectual and Developmental Disabilities Research Center at Baylor College of Medicine. Cells were grown in RPMI 1640 medium containing L-glutamine (Invitrogen) and 10% FBS (Invitrogen).

#### *Array comparative genomic hybridization*

Custom-designed array comparative genomic hybridization (aCGH) version 8.1, also containing probes for single nucleotide polymorphism (SNP) analysis, was performed by the Baylor College of Medicine Medical Genetics Laboratories (MGL, http://www.bcm.edu/geneticlabs/) (Agilent Technologies, Inc., Santa Clara, CA) [13-15]. In addition, a high-density custom tiling-path oligonucleotide microarray spanning the *SBDS* gene and flanking regions [(hg19): chr7:66,422,690-66,480,588] was designed using eArray (http://earray.chem.agilent.com/earray/). The average coverage was 1 probe per 391 bp. Probe labeling and hybridization were performed according to the manufacturer’s protocols with modifications as described [16].

#### *Illumina library construction and exome capture*

Genomic DNA samples were constructed into Illumina paired-end libraries and processed through exome capture according to the manufacturer’s specifications with modifications as described in the BCM-HGSC Illumina Non-Barcoded Paired-End Capture Library Preparation protocol (https://www.hgsc.bcm.edu/sites/default/files/documents/BCM-HGSC_Illumina_Non-Barcoded_Paired-End_Capture_Library_Preparation.pdf).

Pre-capture libraries were prepared using Beckman robotic workstations (Biomek NXp and FXp models). Briefly, 5 μg of genomic DNA in 50 μl volume was sheared into fragments of approximately 400 bp in a Covaris plate with E210 system (Covaris, Inc. Woburn, MA) followed by end-repair, A-tailing and ligation of the Illumina non-barcoded PE adaptors. Pre-capture ligation mediated-PCR (LM-PCR) was performed for 7 cycles of amplification using the 2X SOLiD Library High Fidelity Amplification Mix (a custom product manufactured by Invitrogen). Purification was performed with Agencourt AMPure XP beads after enzymatic reactions. Following the final XP beads purification, quantification and size distribution of the pre-capture LM-PCR product was determined using the LabChip GX electrophoresis system (PerkinElmer).

For exome capture, pre-capture libraries (~1 μg) were individually hybridized in solution to the SeqCap EZ Exome 2.0 design (44 Mb, NimbleGen). Human COT1 DNA and 3’-ddC modified hybridization enhancing oligonucleotides were added into the hybridization to block repetitive genomic sequences and the adaptor sequences. Post-capture LM-PCR amplification was performed using the 2X SOLiD Library High Fidelity Amplification Mix with 14 cycles of amplification. After the final AMPure XP bead purification, quantity and size of the capture library was analyzed using the Agilent Bioanalyzer 2100 DNA Chip 7500. The efficiency of the capture was evaluated by performing a qPCR-based quality check on the four standard NimbleGen internal controls. Successful enrichment of the capture libraries was estimated to range from a 6 to 9 of ΔCt value over the non-enriched samples.

#### *Illumina sequencing*

Library templates were prepared for sequencing using Illumina’s cBot cluster generation system with TruSeq PE Cluster Generation Kits (Part No. PE-401-3001). Briefly, these libraries were denatured with sodium hydroxide and diluted to 3 pM in hybridization buffer in order to achieve a load density of ~800 K clusters/mm^2^. Each library was loaded in a single lane of a HiSeq flow cell, and each lane was spiked with 2% phiX control library for run quality control. The sample libraries then underwent bridge amplification to form clonal clusters, followed by hybridization with the sequencing primer. Sequencing runs were performed in paired-end mode using the Illumina HiSeq 2000 platform. Using the TruSeq SBS Kits (Part No. FC-401-3001), sequencing-by-synthesis reactions were extended for 101 cycles from each end. Sequencing runs generated approximately 200 million successful reads on each lane of a flow cell, yielding an average of 19 Gb per sample. With these sequencing yields, samples achieved an average of 91% of the targeted exome bases covered to a depth of 20X or greater.

#### *Data analysis and variant calling*

Illumina sequence analysis was performed using the HGSC Mercury analysis pipeline (https://www.hgsc.bcm.edu/software/mercury). First, the primary analysis software on the instrument produces .bcl files that are transferred off-instrument into the HGSC analysis infrastructure by the HiSeq Real-time Analysis module. Once the run is complete and all .bcl files are transferred, Mercury runs the vendor’s primary analysis software (CASAVA), which generates sequence reads and base-call confidence values (qualities). The next step is the mapping of reads to the NCBI36 Human reference genome (http://www.ncbi.nlm.nih.gov/projects/genome/assembly/grc/human/) using the Burrows-Wheeler aligner (BWA [17], http://bio-bwa.sourceforge.net/) and producing a BAM [18] (binary alignment/map) file. The third step utilizes GATK [19] (http://www.broadinstitute.org/gatk/) for quality recalibration and also includes BAM sorting, duplicate read marking, and realignment to improve in/del discovery. Next, the Atlas2 [20] suite (Atlas-SNP and Atlas-indel) is used to call variants and produce a variant call file (VCF [21]). Finally, annotation data is added to the VCF using a suite of annotation tools “Cassandra” [22] that brings together frequency, function, and other relevant information using AnnoVar with UCSC and RefSeq gene models, as well as a host of other internal and external data resources.

#### *Probe design and Southern blot hybridization*

A DNA probe was amplified by PCR from BAC clone RP11-347P19 (product size: 322 bp) using primers SBDS_KPN_F: 5’CCCATGCCAATCATTTCTCT3’ and SBDS_KPN_R: 5’GAGACGCACCGAGCTACC 3’, which target the 5’ flanking region of the gene *SBDS*. Genomic DNA was digested with *Kpn*I or *Xba*I (New England BioLabs, Ipswich, MA) for 1 day at 37°C, followed by separation on a 0.7% agarose gel in 0.5X Tris–Borate–EDTA buffer. Hybridization was performed as described [23].

#### *RNA extraction and cDNA preparation*

Total RNA was extracted from LCLs using TRIzol reagent (Invitrogen Corp., Carlsbad, CA); this was DNaseI treated and purified using the RNeasy mini kit according to the manufacturer’s protocol (Qiagen, Valencia, CA). cDNA was synthesized from 1 μg of RNA using qScript cDNA Super Mix (Quanta Biosciences, Gaithersburg, MD).

#### *Short- and long-range PCR amplification, PCR digestion and Sanger sequencing*

Each of the five *SBDS* exons were amplified using primers described in Woloszynek, et al. [11], except for exon 3 which was amplified using primers exon3f1 5’ GATTGTAGTGAGCCGAGATCATACT 3’ and exon3r1 5’CTCCATCCAGTTACTCATTTTTTATG 3’. The amplicons were Sanger sequenced in both forward and reverse directions.

To assay for the presence of short insertions or deletions in the *SBDS* gene or flanking regions, we used primer Del_Fb 5’GTGTCAATTTTCCCCATGCT3’ in combination with primer SBDS_KPN_R to produce a 13.1 kb PCR product, which spans the entire *SBDS* gene plus flanking regions [(hg19): chr7:66,448,791-66,461,897]. *SBDSP1* is not amplified using those primers. The long-range PCR was performed using TaKaRa LA Taq (Clontech, Mountain View, CA). Long-range PCR products were digested using restriction enzymes *Kpn*I and *Sac*I (New England BioLabs, Ipswich, MA) for 2 hours at 37°C, followed by separation on a 1% agarose gel in 0.5X Tris–Borate–EDTA buffer. Amplification of the *SBDS* transcript was performed using cDNA obtained as described above and primers SBDS_3utr_F1 5’GCAGCATGTTCAATGAAAGGTAA3’ and SBDS_5utr_R1 5’ CCTGCCAGACACACTGTGA3’ to generate a 1.4 kb PCR product, which was sequenced by bidirectional Sanger sequencing.

#### *Extraction of protein and western blot analysis*

Lymphoblastoid cells were harvested at a density of approximately 5×10^5^ cells/mL, re-suspended in 200 μL of RIPA buffer + 1% Set III protease inhibitors (VWR) and 1% PMSF (Sigma) and incubated for 30 minutes on ice. The lysate was collected by centrifugation at 21,000 g for 30 minutes at 4°C. Fifty μg of lysate was fractionated by 10% SDS-PAGE. Following transfer to nitrocellulose membrane, the SBDS protein was detected using rabbit α-SBDS antibody (Abcam catalog# ab128946) at 1:5,000 dilution and α-rabbit IgG-IR800 secondary antibody (Li-Cor catalog# 926–32211) at 1:5,000 dilution. The membrane was also probed for β-actin using α-β-actin antibody (Sigma cat# A5441) at 1:5000 dilution and α-mouse IgG-IR800 secondary (Li-Cor Cat# 926–32210) at 1:5,000 dilution.

### Results

#### *Clinical report*

The patient was a white/Hispanic female who was previously evaluated at age 4 years by our immunology and genetics services for recurrent infections, short stature and skeletal abnormalities. She was noted to have the unusual finding of normal serum immunoglobulins with a virtual absence of circulating B cells. Additional clinical features were suggestive of Shwachman Diamond syndrome, although it was noted she carried only a single missense change, which was not previously reported and possibly deleterious, in the *SBDS* gene. Her case was described in a brief report [24]. Here, we elaborate on her clinical features and clinical course and further explore an underlying molecular etiology.

In addition to her evaluations by the immunology and genetics services, she was referred to our hematology center at age 4 years for progressive pancytopenia, with macrocytic anemia. Her absolute neutrophil count fluctuated between <100 to >1,500 cells/μl over a period of 2 ½ years prior to initiating granulocyte-colony stimulating factor (G-CSF). Her bone marrow showed maturing trilineage hematopoiesis with moderate to, eventually, marked hypocellularity (10% cellularity). Additional evaluations demonstrated an elevated hemoglobin F (4.9%), chromosome breakage studies with mitomycin C and diepoxybutane (DEB) within the normal range, and lymphocyte and granulocyte telomere lengths at ~ 40th and <1st percentiles for age, respectively. She became both red blood cell and platelet transfusion-dependent and remained neutropenic despite administration of G-CSF at a dose of 10 μg/kg/day, eventually developing a pseudomonal soft tissue abscess while on G-CSF. She underwent bone marrow transplantation (BMT) for her bone marrow failure with an histocompatibility locus antigen (HLA)-identical sibling donor using a reducing intensity conditioning regimen and remained engrafted without significant transplant-related complications 2 ½ years after BMT, the time of this manuscript preparation.

**Figure 1:**
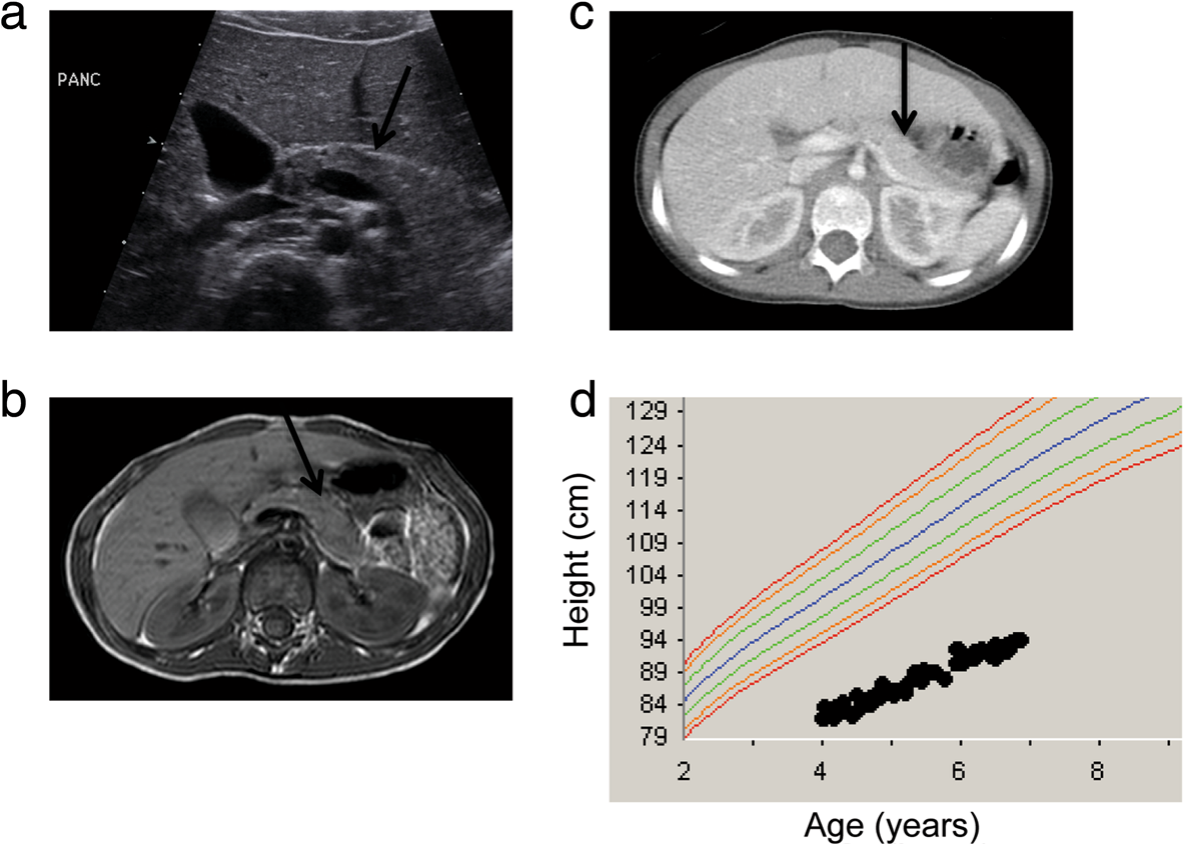
**Clinical features of SDS proband. a**. Transverse abdominal ultrasound image obtained at 5 years 11 months of age revealing normal size, contour, and echogenicity of the pancreas, indicated by arrow. **b**. Axial conventional spin echo T1-weighted MR image obtained at 5 years 11 months of age showing normal signal intensity of the pancreatic parenchyma with no evidence of atrophy or fatty infiltration of the pancreas, indicated by arrow. **c**. Axial contrast-enhanced CT image obtained at 6 years 5 months of age demonstrating normal attenuation of the pancreatic parenchyma with no evidence of atrophy or fatty infiltration of the pancreas, indicated by arrow. **d**. The proband’s growth curve showing markedly short stature. The bottom-most curve normal curve represents the 5th percentile for girls.

The patient had no history of diarrhea or steatorrhea; however, her serum pancreatic isoamylase (ascertained at age 6 years at The Hospital for Sick Children, Toronto) was below the age-adjusted normal range (7 U/L, lower limits of normal 13 U/L), consistent with an SDS pancreatic exocrine deficiency state. Her trypsinogen (93.2 ng/ml) was above normal limits for age (46.5 ng/ml). Despite these findings, her pancreas appeared normal by ultrasound, computed tomography (CT) and magnetic resonance imaging (MRI) (Figure 1a–c), when last obtained at age approximately 6 years. This is an unusual observation, since pancreatic lipomatosis is typically demonstrated by imaging in patients with SDS [25]. In addition, she manifested hyperlipasemia without symptoms of acute or chronic pancreatitis, possibly secondary to macrolipasemia.

Her medical history was also notable for a full term delivery with intrauterine growth retardation (birth weight 2.24 kg), a prolonged postnatal admission for poor feeding, global developmental delay, extreme short stature (<1st percentile) (Figure 1d), marked pectus carinatum [24], metaphyseal chondrodysplasia, tracheomalacia with recurrent lower respiratory tract infections and acute otitis media, severe myopia and chronic hypomagnesemia. Her parents each reported being healthy. She had three healthy full siblings and a sibling stillborn at 5 months gestation.

#### *Molecular genetics*

A diagnosis of SDS was rendered based on established clinical criteria of (1) a hematological cytopenia of any given lineage on at least 2 occasions over at least 3 months and (2) exocrine pancreas dysfunction [26]. Bone abnormalities supported the diagnosis. Her *SBDS* sequence analysis revealed a novel, heterozygous missense variant in *SBDS* exon 1, c.98A > C (p.K33T), mapping to the N-terminal domain (domain I); a region in which most of the *SBDS* disease-causing mutations map [12]. Although this particular variant was novel, a mutation altering the same codon, c.97A > G (p.K33E), was previously reported in a patient with SDS [12]. The mapping of the p.K33E mutation onto the *Archaeoglobus fulgidus* SBDS protein orthologue crystal structure led to the prediction that it altered surface epitopes [12]. Furthermore, cells from a SDS patient carrying the p.K33E mutant protein had altered ribosomal profiles and impaired association of the 40S and 60S subunits; causality was further demonstrated by *in vitro* studies of ribosomal subunit joining using cells expressing the p.K33E variant [9]. Thus, the c.98A > C (p.K33T) variant detected in our patient was deemed likely deleterious. Notably, however, c.98A > C (p.K33T) was the only *SBDS* variant detected.

The inability to identify biallelic mutations at the *SBDS* locus prompted us to explore potentially causative genetic and genomic variation for her disease. Array comparative genomic hybridization with SNP analysis was performed at Baylor College of Medicine Medical Genetics Laboratories as a clinical diagnostic evaluation using a custom-designed array and revealed no copy number variation (CNV) or regions of copy neutral absence of heterozygosity (AOH). Whole exome sequencing of peripheral blood DNA from the proband/parental trio confirmed the presence of the heterozygous missense mutation in *SBDS* exon 1, c.98A > C (p.K33T) and established that it was inherited from the mother. Inspection for deleterious novel or rare single nucleotide variants that were *de novo* or inherited from the father failed to yield any potential variants in candidates that might potentially impact ribosome biology and, therefore, might result in a condition due to digenic or oligogenic inheritance.

**Figure 2:**
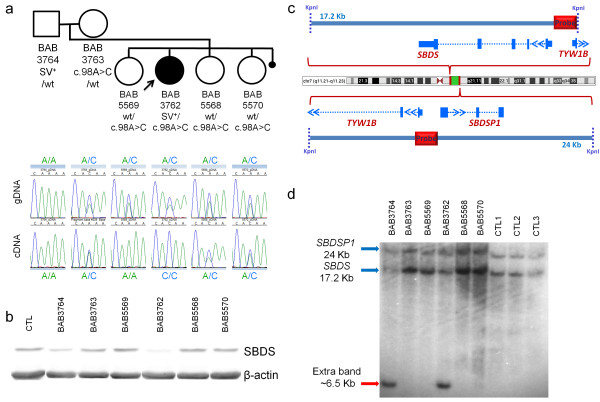
**Segregation of private mutations spanning *SBDS *in family HOU1479.** Missense mutation c.98A > C was maternally inherited by all four children whereas the SV is present only in the father and the patient with SDS. **a**. Top: Pedigree of family HOU1479 with information about carrier status of both mutations in each family member. Bottom: *SBDS* allele status with corresponding chromatograms of exon 1 Sanger sequenced using genomic DNA (gDNA) or cDNA prepared from RNA extracted from LCLs. **b**. Western blot analysis of SBDS in LCL whole cell extracts prepared from an unrelated, unaffected control (CTL), the proband and her family members as indicated. Beta-actin blotting was performed for a protein loading control. **c**. Genomic organization of *SBDS* and *SBDSP1* with identification of the region of the probe used for Southern blot in **d**. **d**. Southern blotting of *SBDS* and *SBDSP1* in samples from family HOU1479 and three unrelated, unaffected controls (CTL1-3) using *Kpn*I restriction enzyme.

Sanger sequencing of the three unaffected siblings indicated that they, too, inherited the missense mutant allele from the mother and analysis of *SBDS* cDNA derived from EBV-transformed LCLs demonstrated that the missense mutant allele was expressed in all but one of the carrier family members (Figure 2a). Remarkably, one of the carrier sister’s (BAB5569) LCLs expressed exclusively the paternally inherited allele, whereas the proband’s (BAB3762) LCLs expressed exclusively the maternally inherited allele, in this assay on clonally derived cells (Figure 2a). These results are consistent with prior reports indicating that the *SBDS* locus might be subjected to differential allele expression in LCLs [27].To determine if the differential allele expression would lead to altered levels of the SBDS protein, we analyzed SBDS levels in the family’s LCLs by western blotting. Notably, both the unaffected father (BAB3764) and the proband’s cells’ SBDS levels were decreased relative to the other family members, including sibling BAB5569, all of which demonstrated levels of SBDS protein similar to each other and an unrelated normal wild type control (Figure 2b).

The differences in the SBDS protein levels between the mother and siblings, who are heterozygous for the p.K33T mutation, and the father and the proband led us to suspect that the proband and father shared an additional *SBDS* molecular alteration not yet revealed by the aforementioned methods. To explore this possibility further, we carried out a variety of molecular approaches: i) additional aCGH, employing a customized high-resolution array spanning the *SBDS* gene and flanking regions, to detect large deletions or duplications; ii) Southern blotting to interrogate the *SBDS* locus; iii) long-range PCR specific for the *SBDS* gene and flanking regions (PCR product of 13.1 kb) followed by digestion using *Kpn*I and *Sac*I in order to detect abnormal size bands relative to samples without the rearrangement; and iv) short-range PCR followed by Sanger sequencing of the *SBDS* exons to detect small deletions and duplications or insertions; all approaches which might uncover genomic rearrangements or SVs.

**Figure 3:**
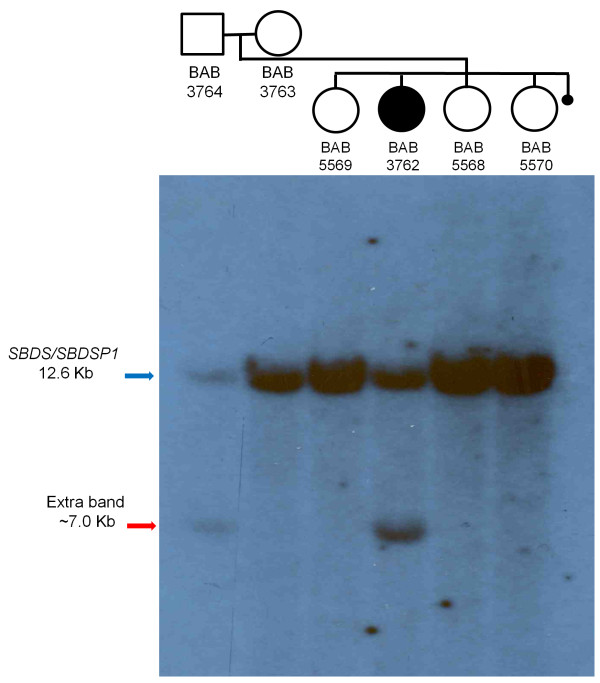
**Southern blotting of *SBDS *** **and *SBDSP1 *** **in samples from family HOU1479 using *Xba*** **I restriction enzyme.**

High-resolution aCGH of the *SBDS* gene showed no evidence of CNV. Remarkably, however, Southern blot analysis using *Kpn*I and a probe specific for the 5’ intergenic region of *SBDS* (Figure 2c) revealed an additional band, approximately 6.5 kb in length, shared by the father and proband and not present in any of the other family members or 11 additional controls (Figure 2d and data not shown). In addition, the father and proband’s samples demonstrated a relative reduction in the *SBDS* 17.2 kb signal (Figure 2d). The presence of a genomic rearrangement, likely an insertion of unknown genomic sequence rather than a point mutation, was supported by additional Southern blotting using a different restriction enzyme (*Xba*I) which produced an extra band of ~ 7.0 kb in both the father and proband (Figure 3).

**Figure 4:**
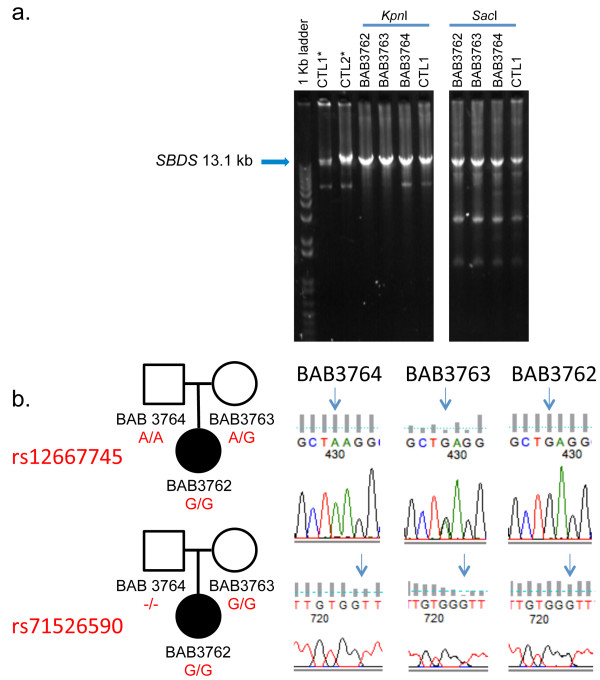
**Additional studies of the genomic segment that includes *SBDS *supports the presence of an insertion of unknown origin within the paternal allele. a**. Agarose gel analysis of a 13.1 kb long-range PCR product spanning *SBDS* and flanking region, the region targeted by the Southern blotting studies (Figures 2 and 3). DNA from patient (BAB3762), parents (BAB3763- mother, BAB3764-father) and a normal controls (CTL1 and CTL2) were amplified using primers DelFb + KpnR, and then digested with either *Kpn*I or *Sac*I. *Kpn*I did not digest the 13.1 kb PCR product, consistent with lack of amplification of the paternal allele. Consistently, *Sac*I digestion of the 13.1 kb PCR product showed an identical pattern in samples and controls. **b**. Sanger sequencing of intron 2 amplified along with exon 2 using short range PCR revealed inconsistent segregation of a paternal genotype for two polymorphic SNPs in the patient (BAB3762). *Non-digested PCR product.

Short- and long-range PCR helped to delineate the nature of such an insertion further. Both approaches failed to reveal band sizes different in the patient and paternal samples from those expected (Figure 4a and data not shown). In addition, digestion of the long-range PCR product of 13.1 kb that includes *SBDS* with *Kpn*I and *Sac*I showed a similar pattern in all samples (Figure 4a). However, in contrast to the detection of both maternal and paternal polymorphic SNPs in the 5’ untranslated region and introns 1 and 3, short range PCR and Sanger sequencing failed to demonstrate the paternal genotype in the patient (BAB3762) for two polymorphic SNPs in intron 2 (Figure 4b). Taken together, these results are consistent with a lack of PCR amplification of the paternal allele that had been revealed by Southern blotting (Figure 4b) due to an insertion resulting in an amplification product larger than what could be afforded by the DNA polymerases used here (data not shown).

## Discussion

**Figure 5:**
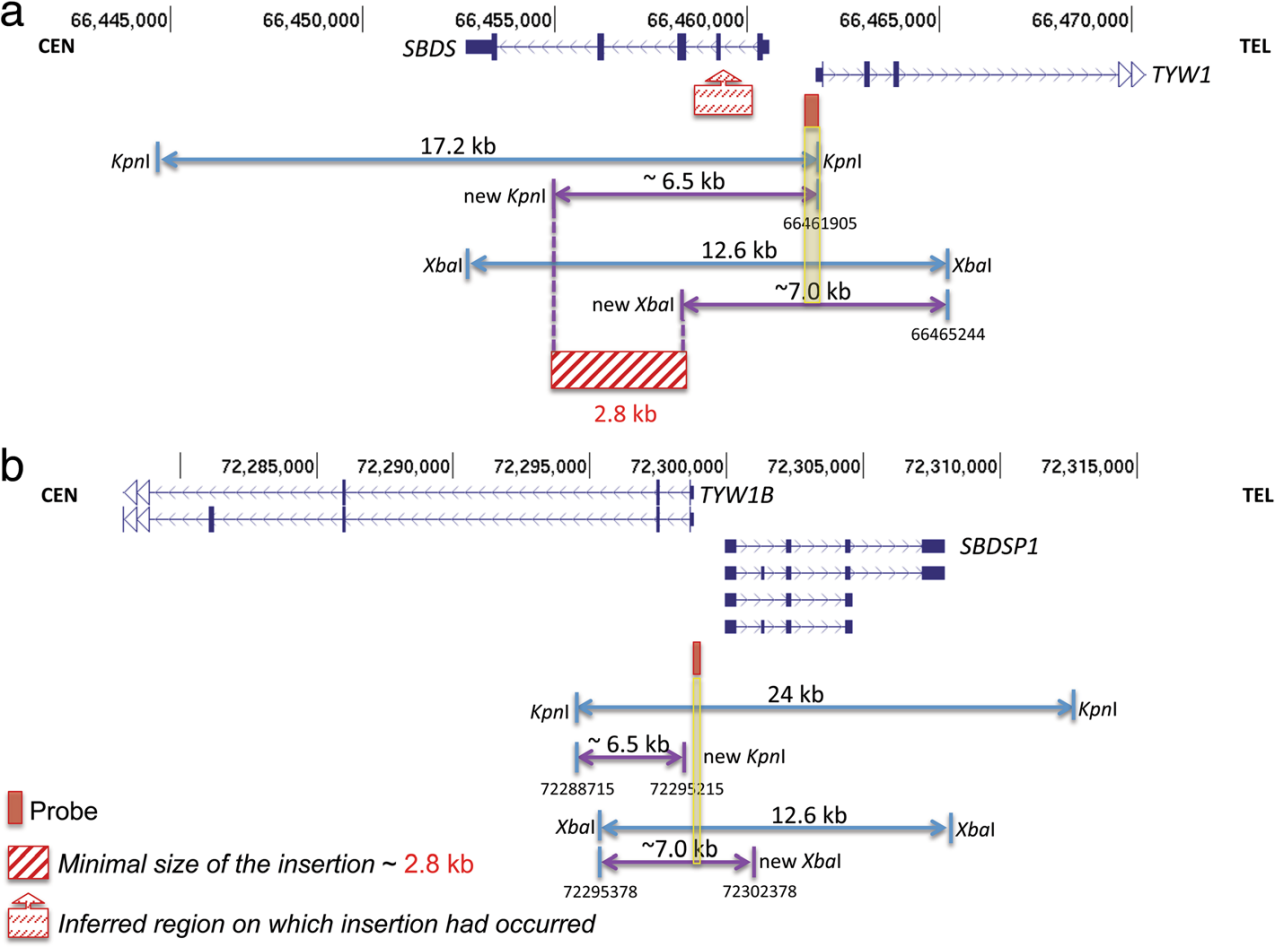
**Approximate location of the inherited SV at the *SBDS *locus based on Southern blotting assays using *Kpn*I and *Xba*I restriction enzymes. a**. Size and location of the expected segments obtained using *Kpn*I and *Xba*I restriction enzymes based on the reference genome (blue arrows) and extra segments obtained exclusively in SV carriers (BAB3762 and BAB3764 - purple arrows). The nature of the SV is currently unknown although the Southern blotting results are consistent with a genomic insertion of at least 2.8 kb (represented by a crosshatch red box) somewhere between *SBDS* 5’flanking region and intron 3 (represented by a red box with an arrow on top). Red box: Southern blotting probe. Yellow rectangle highlights a common probe target region between the reference genome and the genome of the SV carriers. **b**. An alternative hypothesis of SV present within the pseudogene. Genomic coordinates as of GRCh37/ hg19 to *SBDS* and *SBDSP1* loci are represented on top of each region. CEN: centromere; TEL: telomere.

Our findings implicate a genomic rearrangement at the *SBDS* locus in the pathogenesis of the SDS phenotype in our patient and extend the underlying molecular genetic causes of this disease. The molecular nature of the putative genomic alteration was not determined by our study, but we speculate that it is likely a DNA insertion of at least 2.8 kb that introduced new *Kpn*I and *Xba*I sites distal to the probe annealing site (Figure 5a). Insertion of genomic segments can account for at least 42% of SVs, as observed in eight individual genomes cloned in fosmid libraries and subjected to the paired end sequence approach [28]; 20% of those selected for complete sequencing result from retrotranspositional events [28]. Retrotransposition along with insertional translocation events are especially challenging to uncover in CNV studies due to the current lack of appropriate technical tools for high-throughput screening. Additional difficulty is provided by insertion of sequences within low-copy repeat (LCR) or segmental duplications regions, which can be associated with as many as 71% of the CNVs present in an individual genome [29]. Here, we speculate that an unknown segment is inserted in the genomic interval demarcated by the 5’ flanking region to intron 1 or 2 of the *SBDS* locus (Figure 5a), which results in a null allele or leads to an altered regulation of *SBDS*. Both of the latter alternatives are consistent with the decrease in protein expression experimentally observed in the LCLs from the carriers reported herein. Whereas an SV present within the pseudogene *SBDSP1* locus can not be ruled out at this point, it is less likely because there is no common probe target region with the band size obtained upon genomic digestion with *Kpn*I (Figure 5b).

## Conclusions

Our findings lead us to hypothesize that genomic rearrangements contribute to the underlying molecular cause of some cases of SDS without biallelic *SBDS* mutations. In addition to insertion of genomic segments, other structural alterations such as large deletions, duplications or inversions are anticipated due to the peculiar genomic architecture of the *SBDS*/*SBDSP1* loci. Each of the loci resides embedded in a pair of inverted-oriented LCRs consisting of ~301 kb and ~271 kb, respectively, that map ~5.8 Mb apart; the presence of such large LCRs may render the 7q11.21 region prone to instability [5,30]. For instance, intrachromosomal inverted-oriented LCRs may be substrates for NAHR, which can result in segmental inversion of the genomic region in between the LCRs, as exemplified by the H1 and H2 inverted haplotypes at the disease-associated 17q21.31 region [31-33] and recombinant/hybrid LCRs. We predicted previously that formation of hybrid LCRs by NAHR using gene and pseudogenes as substrates could lead to disruption of important disease-associated genes including *SBDS*[30]. Indeed, NAHR between inverted LCRs present within the *IDS* gene and its telomeric pseudogene *IDSP1* is responsible for approximately 13% of mucopolysaccharidosis type II (Hunter syndrome; MIM# 309900) cases [34] and inversion between inverted-oriented LCRs disrupting the factor VIII gene (F8) accounts for >45% of severe hemophilia A (MIM# 306700) cases. Similar events (inversions, large deletions and duplications) have not been reported in SDS patients thus far; we speculate this is likely due to the challenge of studying genes embedded within LCRs. Currently, it is estimated that up to 25% of patients with SDS do not carry *SBDS* biallelic mutations [3,4], therefore, a search for genomic rearrangements within that locus may help unveil an important but underappreciated contributor to this disease.

## Competing interests

JRL holds stock ownership in 23andMe, Inc. and Ion Torrent Systems, Inc., and is a co-inventor on multiple United States and European patents related to molecular diagnostics. MB is founder and chief executive officer of Codified Genomics. All other authors have no conflicts of interest. The Department of Molecular and Human Genetics at Baylor College of Medicine derives revenue from molecular genetic testing offered in the Medical Genetics Laboratories (http://www.bcm.edu/geneticlabs/).

## Authors’ contributions

CMBC conducted high-density CGH arrays, long-range PCR, RT-PCR, experimental design and data analysis. LWZ assisted with long-range PCR, RT-PCR, data analysis and conducted Southern blotting assays. NJN assisted with high-density CGH arrays, long-range PCR, RT-PCR. SJ, DMM and RAG conducted WES experiments, DM and MB performed genomic sequence data analysis. CLW conducted western and Southern blotting assays, assisted with sample collection and data analysis. WI performed the pancreatic isoamylase and trypsinogen assays. RPG interpreted the diagnostic imaging. JRL was involved in research design and data analyses. AAB coordinated clinical studies and subject recruitment, research design and data analyses. CMBC, JRL and AAB prepared the manuscript. All authors read and approved the final manuscript.

## Pre-publication history

The pre-publication history for this paper can be accessed here:

http://www.biomedcentral.com/1471-2350/15/64/prepub
